# Impact of Cattle Breed in scRNA-Seq Reference on Muscle Fiber Type Deconvolution from Bulk RNA-Seq: A Comparison of Software Tools

**DOI:** 10.3390/biotech14030056

**Published:** 2025-07-25

**Authors:** Raphael P. Moreira, Marcelo R. Vicari, Henrique A. Mulim, Theresa M. Casey, Jacquelyn Boerman, Xing Fu, Hinayah R. Oliveira

**Affiliations:** 1 Graduate Program in Genetics, Federal University of Paraná, Curitiba 81531-980, PR, Brazil; rmoreira@purdue.edu; 2 Department of Animal Sciences, Purdue University, West Lafayette, IN 47907, USA; hmulim@purdue.edu (H.A.M.); theresa-casey@purdue.edu (T.M.C.); jboerma@purdue.edu (J.B.); 3 Department of Structural Biology, Molecular and Genetics, State University of Ponta Grossa, Ponta Grossa 84030-000, PR, Brazil; vicarimr@yahoo.com.br; 4 School of Animal Science, Louisiana State University Agricultural Center, Baton Rouge, LA 70803, USA; xfu1@agcenter.lsu.edu

**Keywords:** Brahman, Holstein, *Longissimus dorsi*, myofiber composition, transcriptome, Wagyu

## Abstract

While bulk RNA sequencing provides a comprehensive view of transcriptomes, it lacks cell type specificity. Single-cell RNA sequencing (scRNA-seq) overcomes this limitation by providing detailed insights at the individual cell level, though it involves higher costs. Deconvolution methods can estimate cell type proportions in bulk RNA-seq data, but their results may vary based on the scRNA-seq reference data and software used. This study investigates the estimation of muscle fiber type proportions through deconvolution analysis of *Longissimus dorsi* muscle bulk RNA-seq data from late-gestation Holstein Friesian multiparous cows. Four software tools (i.e., CIBERSORTx, Cellanneal, DeconvR-NNLS, and DeconvR-RLM) were compared using scRNA-seq reference data from Brahman and Wagyu cattle breeds, which included proportions of types I, IIa, and IIx myofibers. Kruskal–Wallis and Dunn’s tests revealed that the breed of reference data significantly influenced the proportions of type IIa and IIx muscle fibers across different deconvolution methods. To the best of our knowledge, this is the first study to show that the cattle breed used in reference scRNA-seq data can substantially impact deconvolution outcomes, highlighting a critical consideration for accurate cell type proportion estimation in livestock genomics. These findings suggest that future deconvolution studies should carefully consider breed compatibility between reference and target datasets.

## 1. Introduction

RNA sequencing (RNA-seq) has revolutionized the analysis of transcriptomes, with bulk RNA-seq enabling analysis of differential gene expression at the global level. Although RNA-seq is a straightforward and cost-effective technique, it cannot fully capture the intrinsic heterogeneity of samples to reveal the contribution of different cell types and states of development to global gene expression [1,2,3]. Single-cell RNA-seq (scRNA-seq), on the other hand, provides cell-type-specific transcriptome profiling, enabling the determination of cellular composition and proportions. However, the relatively high cost of scRNA-seq and technical challenges such as tissue dissociation and preparation of single-cell or nuclear suspensions limits its applicability, especially when working with archived or previously collected samples [4,5]. The recent emergence of computational deconvolution methods provides cost-effective alternatives to estimate the proportions of different cell types from bulk RNA-seq data [2]. These approaches infer the cellular composition of heterogeneous tissues by mathematically decomposing bulk gene expression profiles, often using reference signatures derived from scRNA-seq data. Depending on the algorithm, deconvolution can be performed through linear regression, support vector regression, or probabilistic modeling, allowing researchers to estimate relative contributions of individual cell types without requiring single-cell data generation [6].

Various software tools are currently available for estimating cell proportions, each differing in reference dataset requirements and statistical methodologies used for deconvolution analysis. Tools that use a reference based on previously generated scRNA-seq data generally provide more reliable estimates of cell proportions compared to those that do not use a reference dataset [3]. Among reference-based tools, CIBERSORTx [7] is a commonly used deconvolution tool that applies a support vector regression algorithm to gene expression data for the estimation of cell type proportions. CIBERSORTx is particularly effective for analyzing complex heterogeneous samples [3,8]. Similarly, Cellanneal [9] is a deconvolution method that uses simulated annealing to optimize Spearman’s rank correlation between experimental and computational gene expression vectors. By ranking gene expression values, Cellanneal reduces the influence of highly expressed genes and incorporates a broad set of informative genes, minimizing dependency on a specific gene list [9]. DeconvR [10] offers multiple methods for deconvolving bulk RNA-seq data, including non-negative least squares (NNLS) and robust linear regression (RLM). NNLS [11] minimizes the sum of squared residuals while ensuring that all estimated proportions remain non-negative, making it suitable for datasets with non-negative constraints, such as gene expression levels or proportions of cell types. On the other hand, RLM is more resilient to outliers, as it minimizes the influence of extreme values, providing robust estimates when the data may contain noise or large variability [10].

Despite its potential, deconvolution analyses remain underutilized in studies focused on livestock animals. A major challenge lies in the limited availability of reference data for specific tissues, compounded by the fact that existing scRNA-seq datasets are often derived from diverse breeds within the same species. Most available studies focus on humans and mice, leaving gaps in understanding how deconvolution performs across livestock breeds. In cattle, for instance, only one study reporting scRNA-seq analysis of bovine skeletal muscle tissue samples is currently publicly available [12]. This study produced scRNA-seq data of skeletal muscle from Wagyu and Brahman cattle, and their crossbreed, and revealed mechanisms contributing to differential intramuscular fibrogenesis and adipogenesis between the breeds [12]. However, there are over 800 recognized cattle breeds, categorized into *Bos taurus* and *Bos indicus* [13]. Although significant opportunities exist in applying deconvolution analysis to bulk RNA-seq data to enhance our understanding of physiology of livestock in general, and cattle in particular, knowledge of how scRNA-seq reference data from different breeds may impact cell type identification is needed.

In dairy cattle, skeletal muscle plays an important role as a protein reserve to support milk production, particularly in early lactation [14]. Mobilization of skeletal muscle in early lactation provides essential energy and amino acids to sustain high milk production [14]. Skeletal muscle of mature cattle is primarily composed of slow myofibers (type I) and fast myofibers (types IIa and IIx) [15]. We recently performed transcriptome analysis of *longissimus dorsi* (LD) muscle biopsied from Holstein Friesian (*Bos taurus*) cows in late gestation and found expression levels of genes that encode for type I, IIa, and IIx myofibers varied between cattle with high LD muscle depth versus low depth at three weeks before expected calving [16]. However, since these studies were conducted using bulk RNA-seq and not scRNA-seq analysis, the proportion of specific cell fractions and their functional implications could not be surmised. A precise characterization of muscle fiber types via deconvolution may offer valuable insights into muscle physiology and its influence on key productive and reproductive traits in dairy cattle.

To address these challenges, the present study aimed to assess the performance of four deconvolution methods—CIBERSORTx (v. 1.05), Cellanneal (v. 1.1.0), DeconvR-NNLS (v. 1.15.0), and DeconvR-RLM (v. 1.15.0) —in estimating the proportions of type I, IIa, and IIx myofibers. This assessment was performed using bulk RNA-seq data from Casey et al. [16], in combination with what is, to the best of our knowledge, the only currently available single-cell RNA-seq reference dataset, derived from two genetically distinct bovine breeds: Wagyu (*Bos taurus*) and Brahman (*Bos indicus*) [12].

Despite the increasing availability of deconvolution tools, it remains unclear how breed-specific differences in single-cell reference data influence the accuracy and consistency of muscle fiber type estimation from bulk RNA-seq in cattle. We hypothesize that using genetically distinct scRNA-seq references may lead to significant differences in the inferred myofiber proportions. Therefore, our objective was to systematically evaluate the consistency and biological relevance of deconvolution outputs generated by each method across different reference panels, aiming to identify the most robust approach for studying muscle composition in dairy cattle.

## 2. Materials and Methods

**Figure 1:**
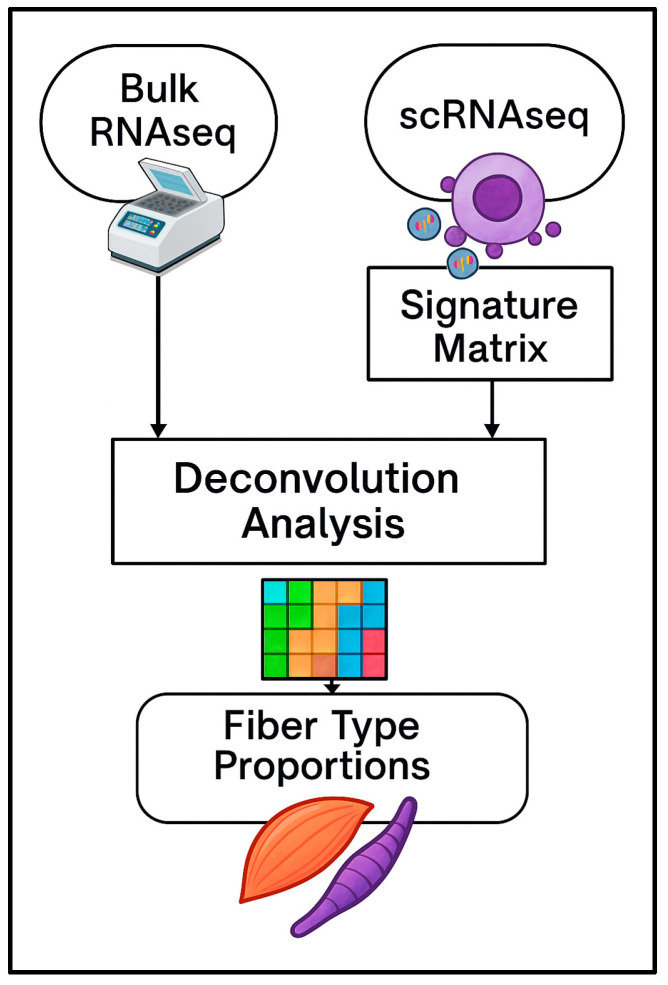
Overview of the computational deconvolution workflow. Bulk RNA-seq data (left) are integrated with a reference single-cell RNA-seq dataset (right), from which a signature matrix is derived. These inputs are used in the deconvolution analysis, resulting in the estimation of cell-type-specific proportions, here represented as muscle fiber types.

Bulk RNA-seq and single-cell RNA-seq data were obtained from publicly available databases (GEO accession numbers GS267667 and GSE205347, respectively). Figure 1 illustrates an overview of deconvolution analysis workflow.

### 2.1. Bulk RNA-Seq

The RNA-seq dataset used in this study was obtained from Casey et al. [16]. Biopsies of the LD muscle had been performed in 39 multiparous Holstein dairy cows three weeks before the expected calving. Samples were sent to Genewiz, Azenta Life Sciences (South Plainfield, NJ, USA) for total RNA isolation and next-generation sequencing using their Standard RNA-Seq Data Analysis Package. Cow selection was based on the sufficient availability of biopsy tissue for analysis. Total RNA was isolated using the RNeasy Plus Universal Mini Kit (Qiagen, Germantown, MD, USA), following the manufacturer’s guidelines. Ribosomal RNA was depleted from the total RNA using QIAseq FastSelect–rRNA HMR kits (Cat. No./ID: 335376, Qiagen). Subsequently, the RNA-sequencing library was prepared according to the manufacturer’s instructions using the NEBNext Ultra II RNA Library Prep Kit for Illumina (NEB, Cat. No. E7775, Ipswich, MA, USA).

The libraries were multiplexed and clustered on two lanes of a flow cell, then sequenced on the Illumina NovaSeq S4 instrument (Illumina, San Diego, CA, USA) in a 2 × 150 paired-end configuration, as directed by the manufacturer. Trimmomatic v.0.36 was used to remove adapter sequences and low-quality nucleotides. On average, each sample yielded 28.3 million reads (±3.4). The mean Illumina quality score (Q score) across all samples was 35.6 (±0.06), indicating the likelihood of a base-calling error; higher scores signify more reliable base calls. Specifically, a Q score of 40 predicts one incorrect base call in 10,000, while a Q score of 30 predicts one incorrect base call in 1000. The resulting data were deposited in the National Center for Biotechnology Information (NCBI) Gene Expression Omnibus (GEO-Accession No. GS267667). The counts were in transcripts per million (TPM). For complete details on the animals and bulk RNA-seq used in this study, please refer to Gouveia et al. [17] and Casey et al. [16].

### 2.2. Reference scRNA-Seq

The scRNA-seq dataset from Wang et al. [12] was used as a reference to deconvolve the bulk RNA-seq data. In summary, Wang et al. [12] performed scRNA-seq on cells isolated from the LD muscle of 4-month-old female calves from three genetic backgrounds: purebred Wagyu, purebred Brahman, and Wagyu/Brahman crossbred. The crossbred dataset was excluded from our study due to the extremely low number of type IIx myofibers (only two cells) in the reference data. This exclusion was based solely on the limited cell representation.

In the study by Wang et al. [12], the muscle tissue was processed by mincing and digesting in Dulbecco’s modified Eagle’s medium (DMEM) with collagenase D and Dispase II, followed by filtration and centrifugation to obtain a cell suspension. Debris and dead cells were removed using specialized solutions from Miltenyi Biotec (North Rhine-Westphalia, Germany). The scRNA-seq libraries were constructed using Chromium Single Cell 3′ Reagent Kits v3 (10X Genomics, Pleasanton, CA, United States) and sequenced on the Illumina NovaSeq platform, achieving over 20,000 read pairs per cell. Raw sequencing data were processed with Cell Ranger 6.0.0 and aligned to the ARS-UCD1.2 bovine genome [18]. The processed data were analyzed using the Seurat package (version 4.0.4) [19] available in the R software v. 4.4.1 [20], where cells with over 5% mitochondrial gene expression were excluded. The genomic sequencing datasets have been deposited in GEO (GSE205347).

To ensure consistency in the deconvolution process, we used the same number of cell types from both breeds. To achieve this, we limited the number of cell types to match the smallest count observed in either breed. Cell types exceeding this number in the other breed were excluded from the dataset. Thus, we retained 5197 distinct cells across the following categories: B lymphocytes, CD3-T cells, CD4-T cells, CD8-T cells, fibroblast-activation-protein-positive cells (FAP-1 and FAP-2), granulocytes (Granulocyte-1), lymphatic endothelial cells (LEndo), monocytes and macrophages (Mo/Ma), mural cells (MUC-1 and MUC-2), natural killer (NK) cells, non-vascular endothelial cells (Non-VEndo), proliferating T cells, satellite cells, and venular endothelial cells (VEndo).

A detailed stratification was used in this study, where slow fibers were categorized as type I (39 cells), and fast fibers were divided into type IIa (29 cells) and type IIx (10 cells). We also limited the number of fibers to match the smallest count observed in either breed. Fibers exceeding this number in the other breed were excluded from the dataset. All datasets were normalized using the LogNormalized method [21]. For additional details regarding the scRNA-seq data, please refer to Wang et al. [12].

### 2.3. Deconvolution Analysis

Four different deconvolution methods available in three software tools were tested: Cellanneal (https://github.com/LiBuchauer/cellanneal.git, accessed on 27 July 2024) [9], CIBERSORTx (https://cibersortx.stanford.edu/, accessed on 2 July 2024) [7], and DeconvR (using both NNLS and RLM methods) (https://github.com/BIMSBbioinfo/deconvR.git, accessed on 12 August 2024) [10]. Deconvolution analysis using type I, IIa, and IIx myofiber classification was performed with each software, using both reference breeds. A brief description of the methods and parameters used in each software is included below.

Signature Matrix: Before proceeding with deconvolution, the first step was to generate a signature matrix from the scRNA-seq data. The signature matrix was created using the scRNA-seq reference for the data containing the type I, IIa, and IIx classification. This matrix contains gene expression profiles from distinct cell types or subtypes, serving as a reference to estimate the contribution of each cell type in mixed samples [8]. The “Create Signature Matrix” option implemented in CIBERSORTx [7] was used for this purpose. The signature matrix was used as an input in all software tested. Default parameters were used with 10 replicates to generate balanced profiles, since this was the maximum number of type IIx myofibers in the Brahman breed. Default parameters recommended by the software developer included a minimum gene expression threshold of 0.75 and random sampling of 50% of available single-cell gene expression profiles (GEPs). Additional settings were a maximum condition number of 999, a q-value cutoff of 0.01, and a barcode gene range of 300–500. The resulting signature matrix was subsequently used as input for all deconvolution analyses.

CIBERSORTx (v. 1.05): The fraction function of CIBERSORTx [7] was accessed via a Docker container (https://hub.docker.com/r/cibersortx/fractions, accessed on 2 July 2024). This software employs support vector regression, a machine-learning approach, to enhance its ability to deconvolute complex gene expression mixtures by selecting relevant features, enabling the resolution of closely related cell subsets. To achieve this, it constructs a signature matrix from pure cell type data and uses recursive feature elimination (RFE) to iteratively identify the most informative genes for distinguishing cell types [7]. CIBERSORTx [7] quantifies the relative proportions of different cell types within bulk tissue samples by leveraging predefined cell-type-specific gene signatures. This requires a matrix of bulk tissue gene expression profiles and a signature matrix containing gene expression profiles unique to each cell subset for accurate deconvolution [8]. To estimate the proportion of a specific cell type, the algorithm evaluates expression changes in the signature genes associated with that cell type relative to all other genes in the sample [22]. CIBERSORTx was applied to estimate cell fractions using the signature matrix and bulk RNA-seq data normalized in TPM log format, with B-mode batch correction and 100 permutations. As recommended by Newman et al. [22], B-mode batch correction is essential for minimizing technical variation between bulk RNA-seq and scRNA-seq datasets.

Cellanneal (v. 1.1.0): The Cellanneal [9] tool uses Spearman’s rank correlation coefficients estimated between synthetic gene expression vectors (which represent modeled profiles from known cell types) and bulk gene expression data as the objective function for its optimization process [23]. This correlation method is advantageous because it relies on ranks rather than raw data values, ensuring that each gene contributes equally to the optimization outcome, regardless of its expression level [23]. By focusing on rank-based correlations, Cellanneal mitigates the influence of outliers and variations in absolute gene expression levels, allowing for a more robust estimation of cell type proportions [9,11,24]. Cellanneal was used to estimate cell fractions, applying the signature matrix generated by CIBERSORTx and bulk RNA-seq data (normalized in TPM log format). The program’s default parameters were used, with minimum and maximum expression levels in the mixture sample set to 1 × 10^−5^ and 0.01, respectively. The minimum scaled dispersion was set to 0.5, and the maximum number of iterations was 1000.

DeconvR (v. 1.15.0): The deconvR package [10] available in R [20], was used based on two different methods: non-negative least squares (NNLS) and robust linear regression (RLM). The NNLS method employs a technique for solving least square problems under the constraint that all solutions must be non-negative, as described by Lawson and Hanson [11]. In contrast, the RLM approach fits a robust linear model, using the expression profiles of different cell types as explanatory variables, with the mixture samples serving as dependent variables, in line with the principles outlined by Wilcox [25]. Both methods of DeconvR were used to estimate cell fractions by applying the signature matrix generated by CIBERSORTx and bulk RNA-seq data (normalized in TPM format). Both methods used the default parameters recommended by the software developers, including a maximum of 500 iterations, a convergence tolerance of 1 × 10^−5^, and normalization of input data enabled by default.

### 2.4. Statistical Analysis Used for the Comparisons

Statistical analyses were performed in R [20] to identify differences among methods, reference breeds, and benchmark biopsies for type I, type IIa, and type IIx myofibers. Outliers in the deconvolution results for all methods (using both reference breeds) were excluded before analysis. A Z-score threshold of less than −3.5 or greater than 3.5 was used as the exclusion criterion. As recommended by Barnett and Lewis [26], removing outliers using Z-scores can further reduce the influence of extreme values, which can violate the assumptions of normal distribution and lead to inaccurate estimates of means and variances.

Descriptive statistics, including minimum, maximum, mean, and standard deviation, were calculated for type I, IIa, and IIx myofibers for each combination of reference breed, software tool, and biopsy. To assess the appropriateness of parametric testing, we first fitted two-way ANOVA models for each fiber type, with the deconvolution method and reference breed as the main factors, including their interaction term. The Shapiro–Wilk test (*p* < 0.05) was performed on the two-way ANOVA residuals using the shapiro.test function in R [27]. Since the two-way ANOVA residuals did not meet the normality assumption (type I: W = 0.965, *p* < 0.001; type IIa: W = 0.974, *p* < 0.001; type IIx: W = 0.967, *p* < 0.001), the non-parametric Kruskal–Wallis test [28] was used instead to compare the distributions of the groups. The analyses were carried out using Dunn’s test, available in the dunn.test R package (version 1.3.5) [29], using Bonferroni multiple testing correction to account for the 28 pairwise comparisons (*p* < 0.00179). This threshold was used to determine statistical significance between the values.

## 3. Results

### 3.1. Deconvolution Analysis Results Across Methods and Reference Breeds

This study demonstrates significant differences in estimated myofiber proportions depending on both the software tool used and the cattle breed employed as reference data. The total myofiber proportion is calculated as the sum of the myofibers in the output, expressed as a proportion relative to other cell types, and outcomes varied substantially across methods and reference breeds. For instance, when Brahman and Wagyu were used as reference breeds for deconvolving Holstein muscle transcriptome RNA-seq, the estimated proportions—representing the percentage of transcriptomic similarity between the target (Holstein) and each reference breed—were as follows: CIBERSORTx estimated 95.21% (±1.31%) and 89.48% (±1.91%), Cellanneal estimated 66.38% (±2.84%) and 71.88% (±2.56%), DeconvR-NNLS estimated 100% (±0.00%) and 99.84% (±0.27%), and DeconvR-RLM estimated 92.76% (±1.75%) and 88.87% (±2.91%), respectively.

### 3.2. Myofiber Proportions

**Figure 2:**
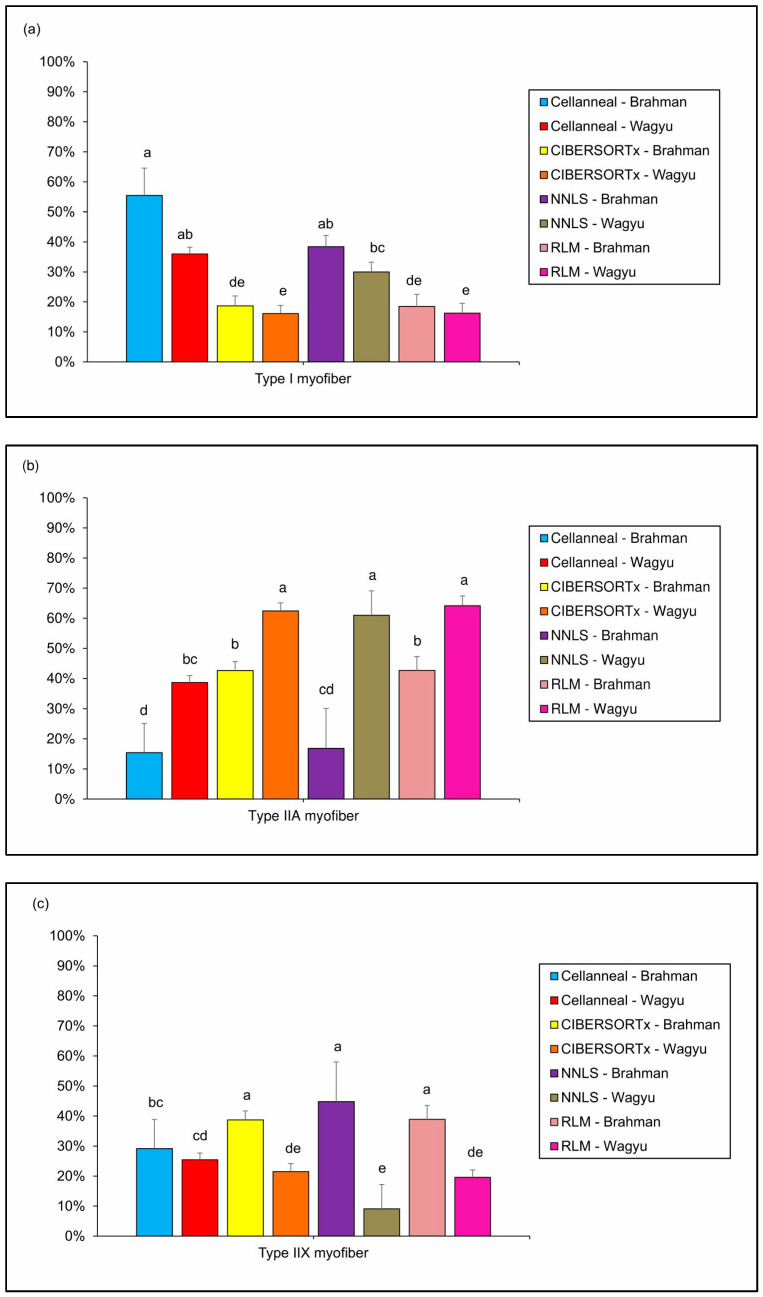
Bar plots showing the mean values of (**a**) type I, (**b**) type IIa, and (**c**) type IIx myofibers from Holstein cows, as estimated by deconvolution using four methods (CIBERSORTx, Cellanneal, DeconvR-NNLS, and DeconvR-RLM), using Brahman and Wagyu as reference breeds. Error bars represent the standard deviation of the means. NNLS—non-negative least squares; RLM—robust linear model. Different letters above the bars indicate statistically significant differences between groups, as determined by Dunn’s test with Bonferroni multiple-test correction (*p* < 0.00179).

For type I myofiber proportions (Figure 2a), all methods produced relatively similar outcomes when using either Brahman or Wagyu reference datasets. The highest type I myofiber proportions were obtained using Cellanneal with both reference breeds (Brahman: 55.14%; Wagyu: 36.01%) and DeconvR-NNLS with Brahman as the reference breed (37.41%). These values were followed by that by DeconvR-NNLS with Wagyu (29.21%). The lowest proportions for type I myofibers were consistently observed with CIBERSORTx and DeconvR-RLM across both reference breeds. For instance, CIBERSORTx reported values of 18.67% for Brahman and 16.09% for Wagyu, while DeconvR-RLM estimated similar values of 17.39% for Brahman and 15.36% for Wagyu.

For type IIa fibers (Figure 2b), the analysis revealed pronounced breed-specific effects that were consistent across multiple deconvolution methods. The highest proportions of type IIa fibers were obtained using Wagyu as the reference breed in CIBERSORTx, DeconvR-NNLS, and DeconvR-RLM, with values of 62.40%, 61.43%, and 64.66%, respectively. These estimates were followed by the proportions obtained with Wagyu in Cellanneal (38.64%) and with Brahman in both CIBERSORTx (42.41%) and DeconvR-RLM (43.64%). Consequently, the lowest proportions of type IIa fibers were obtained when Brahman was used as the reference breed, specifically with Cellanneal (15.89%) and DeconvR-NNLS (19.10%). Across all methods, using Wagyu as the reference consistently resulted in higher proportions of type IIa fibers compared to Brahman.

For type IIx myofibers (Figure 2c), the analysis revealed a pattern opposite to that observed for type IIa fibers. The highest proportions were observed when Brahman was used as the reference breed in CIBERSORTx, DeconvR-NNLS, and DeconvR-RLM, with values of 38.93%, 43.49%, and 38.97%, respectively. Cellanneal produced similar estimates for both reference breeds, with 28.97% for Brahman and 25.15% for Wagyu. The lowest proportions of type IIx myofibers were reported when Wagyu was used as the reference in CIBERSORTx (21.51%), DeconvR-NNLS (9.36%), and DeconvR-RLM (19.97%). Notably, apart from Cellanneal, which provided comparable values for Brahman and Wagyu, all other methodologies consistently reported higher proportions of type IIx myofibers when Brahman was used as the reference breed.

### 3.3. Statistical Analysis and Software Performance Comparison

Kruskal–Wallis tests revealed statistically significant differences in the estimated proportions of type IIa and IIx muscle fibers when comparing different combinations of software tools and reference breeds (*p* < 0.00179 for both fiber types). Subsequent Dunn’s tests with Bonferroni correction identified specific pairwise differences between methods and reference breeds. For type I fibers, no statistically significant differences were observed between reference breeds within the same software tool.

When comparing the performance of different deconvolution methods, CIBERSORTx and DeconvR-RLM demonstrated the most consistent results across different reference breeds, with both methods showing similar sensitivity to breed-specific effects. These methods predicted 90 to 95% of myofibers in the samples. On the other hand, DeconvR-NNLS consistently estimated total myofiber proportions near 100%. Cellanneal showed intermediate performance with more variable results across reference breeds, estimating total myofiber proportions ranging from 66% to 72%.

## 4. Discussion

### 4.1. Novel Breed-Specific Effects in Deconvolution Analysis

The deconvolution analysis of bulk RNA-seq data from Holstein cows found that the breed of cattle used in the reference scRNA-seq data and the software tools applied significantly influenced the predicted myofiber proportions in LD muscle. According to Im and Kim [2], benchmarking is essential for validating deconvolution methods. This is the first study to show that the cattle breed used in single-cell RNA-seq reference data significantly impacts the outcomes of deconvolution analyses for muscle fiber type estimation. Our comprehensive comparison of four deconvolution methods using reference data from two genetically distinct cattle breeds revealed consistent breed-specific effects on the estimation of type IIa and IIx myofiber proportions, while type I fiber estimation remained relatively stable across reference breeds.

The systematic nature of the breed effects observed across multiple deconvolution algorithms suggests that these differences reflect genuine biological variations in gene expression patterns between cattle breeds rather than methodological artifacts. However, without true scRNA-seq data from Holstein cows as ground truth, we cannot definitively determine which breed reference or deconvolution method provides the most accurate estimates of myofiber proportions. This limitation underscores the need for future studies to generate breed-matched single-cell reference data to validate deconvolution approaches and establish definitive benchmarks for accuracy assessment. Interestingly, the consistency of breed-specific effects across CIBERSORTx, DeconvR-NNLS, and DeconvR-RLM for type IIa and IIx fibers, despite their different algorithmic approaches, suggests that breed compatibility between reference and target datasets is a critical factor for accurate deconvolution results. These findings highlight two potential strategies for improving deconvolution accuracy: either using breed-matched reference datasets when analyzing specific populations, or alternatively, developing comprehensive multi-breed reference datasets that capture the full spectrum of genetic diversity within and across species. The latter approach could support OneHealth initiatives by enabling cross-species comparative analyses and leveraging the broader biological knowledge available across different breeds and species to enhance deconvolution robustness.

In domestic animals, the existence of multiple breeds or lineages within a species—each selected for specific traits such as intramuscular fat, growth rate, milk yield, muscle fiber composition, or heat tolerance—leads to distinct gene expression profiles across tissues. These breed-specific transcriptomic differences help explain the systematic effects observed in our deconvolution results, reinforcing the importance of using representative or breed-matched reference datasets for accurate cell type proportion estimation.

### 4.2. Performance Comparison of Deconvolution Methods

CIBERSORTx and DeconvR-RLM demonstrated similar estimates of fiber type distribution across both reference breeds. Additionally, when analyzing segmented myofibers, both methods calculated similar proportions of type I fibers using the different breed reference datasets. In contrast, the estimated proportions of type IIa and IIx myofibers varied depending on the reference breed, suggesting these fiber types may be more sensitive to breed-specific transcriptional differences. CIBERSORTx and DeconvR-RLM were considered the most consistent methods due to their low inter-sample variability and agreement across the reference datasets. Their estimates also aligned with established physiological expectations in bovine skeletal muscle, particularly the predominance of type II fibers and a total myofiber content of approximately 90–95% in muscle samples, which supports the biological plausibility of their outputs. On the other hand, DeconvR-NNLS showed notably poor performance by consistently estimating unrealistic total myofiber proportions near 100%, which contradicts the known cellular heterogeneity in muscle tissue that includes fibro-adipogenic progenitors, satellite cells, and immune cells. This limitation likely stems from the non-negative least squares constraint, which forces the algorithm to assign all transcriptomic signal to the available myofiber types rather than recognizing the presence of other cell populations, thereby undermining the method’s ability to provide biologically accurate estimates of muscle composition.

Performance differences between CIBERSORTx and DeconvR-NNLS may also reflect their underlying algorithmic assumptions, with CIBERSORTx’s support vector regression framework providing greater noise tolerance and adaptive feature selection compared to DeconvR-NNLS’s strict non-negative constraints that limit flexibility in handling crossbreed transcriptomic variability. For instance, Jin and Liu [30], in their evaluation of 11 deconvolution methods across 1766 different conditions in *Homo sapiens*, also demonstrated that CIBERSORTx achieved the best performance. Muscle samples from cows exhibit significant cellular heterogeneity, and during late lactation, gene expression profiles may shift due to physiological changes [16]. Moreover, immune cell populations can increase as part of an inflammatory process before parturition [31]. CIBERSORTx and DeconvR-RLM, which rely on linear regression for deconvolution, seem to perform effectively with heterogeneous tissue samples and manage outliers through Bayesian frameworks or M-estimators [3,7,32]. These factors can make CIBERSORTx and DeconvR-RLM particularly well-suited for accurately predicting transcriptomic signals and identifying myofiber fractions in dynamic biological contexts.

DeconvR-NNLS consistently estimated total myofiber proportions near 100%. However, this assumption is likely incorrect. Wang et al. [12] demonstrated the presence of various cell types, including fibro-adipogenic progenitors (FAPs), satellite cells (SCs), monocytes/macrophages, neutrophils, and lymphocytes, in muscle samples of Wagyu and Brahman cattle. Similarly, Xiao et al. [33], analyzing scRNA-seq data from the LD muscle in pigs, identified endothelial cells, myotubes, FAPs, satellite cells, myoblasts, myocytes, Schwann cells, smooth muscle cells, dendritic cells, pericytes, and neutrophils. Zhu et al. [34] reported similar findings in the LD muscle of goats, describing not only myofibers but also fibroblasts, endothelial cells, satellite cells, and smooth muscle cells. The limitation of DeconvR-NNLS likely arises from a methodological constraint, wherein the algorithm enforces non-negative estimates for proportions to provide a biologically interpretable output. While this constraint avoids negative proportions, it undermines the method’s ability to detect cellular heterogeneity, particularly for cell populations present at low abundance within the analyzed tissue. This methodological bias hampers the accurate representation of the diverse cellular composition typically observed in muscle tissues.

Wang et al. [4] benchmarked four deconvolution methods, including CIBERSORTx and DeconvR-NNLS, in pancreatic islet cells from humans under two distinct scenarios. In the first, both scRNA-seq and bulk RNA-seq data were derived from the same individuals. In this case, DeconvR-NNLS and CIBERSORTx performed comparably, with correlation values of 0.85 and 0.89, respectively. In the second scenario, where scRNA-seq and bulk RNA-seq data came from different individuals, the performance of both methods declined. The correlation for CIBERSORTx dropped more significantly to 0.76, compared to 0.82 for DeconvR-NNLS, indicating a greater decrease in its performance under this condition. However, in our study, despite using a different breed reference to deconvolute the Holstein muscle transcriptome, CIBERSORTx demonstrated more consistent results across different reference breeds compared to DeconvR-NNLS.

### 4.3. Biological Basis for Breed-Specific Expression Patterns

The importance of reference breeds became particularly evident for certain myofiber types (Figure 2b,c). Despite no differences being found between breeds for type I fibers, we observed variability across all methods for type IIa fibers. For type IIx fibers, CIBERSORTx, DeconvR-NNLS, and RLM analyses showed clear discrepancies between reference breeds. Wagyu is well known for its high intramuscular fat (IMF) content [35]. Gotoh et al. [36] reported significantly higher IMF levels in Wagyu compared to Holstein and found a positive correlation between IMF and type I fiber abundance, in contrast to a negative correlation for type II fibers. When using Wagyu as the reference, estimates for type IIa fibers were generally higher and more consistent across methods, suggesting that the transcriptomic profile of Wagyu muscle may enhance the detection or resolution of type-IIa-specific gene signatures. However, for type IIx fibers, Wagyu-based references produced the lowest estimated proportions in CIBERSORTx, DeconvR-NNLS, and RLM analyses. In contrast, Wang et al. (2023) [12] showed that Brahman has a greater fibrogenesis capacity than Wagyu, which may amplify muscle-specific gene expression signals and improve the accuracy of myofiber type deconvolution. Consequently, Brahman outperformed Wagyu as a reference breed when deconvoluting Holstein muscle tissue, particularly for type IIx fibers.

### 4.4. Comparison with the Literature and Validation Context

There is a notable lack of scRNA-seq or single nuclear RNA-seq (snRNA-seq) studies of the fractions of cell types in the LD muscle of cattle. Notably, our findings indicate a higher proportion of muscle cells compared to those documented in the existing literature in the LD of other species such as swine. Yi et al. [37] found that the total muscle myofiber proportions ranged from 64.12% to 73.60% of the cells using snRNA-seq of the LD muscle of Dahe pigs at 194 days of age. Similarly, Wang et al. [25], using snRNA-seq analysis of LD muscle in Laiwu pigs, reported total muscle fiber values of 73.74% and 82.59% of cells. In contrast, Xiao et al. [33], using scRNA-seq of LD muscle from a 1-day-old newborn Suhuai boar, found a lower percentage of total muscle fibers, approximately 28.5%.

Mammalian skeletal muscle typically comprises myofiber isoform types I, IIa, IIb, and IIx [38]. In Casey et al. [16], our bulk RNA-seq dataset, normalized read counts were shown for MYH4, the gene encoding type IIb fibers. However, in our deconvolution study, we identified proportions only for type I, IIa, and IIx myofibers in Holstein cows. This is likely because the reference data used here did not detect type IIb myofibers in Wagyu or Brahman breeds [12], preventing their detection in our results. Other studies have also reported the absence of type IIb myofibers in bovine species, including Holstein [39] and Simmental cattle [15], particularly in the LD muscle.

According to the literature, the proportion of slow myofibers in bovine LD muscle is consistently lower, ranging from 25% to 32.5%, compared to fast myofibers, which range from 67.5% to 75% [40,41]. Similarly, Casey et al. [16], who analyzed bulk RNA-seq data from the same Holstein cows used in our study, reported that expression of MYH7 (which encodes the type I myosin heavy chain) was substantially lower (~13%) compared to the combined expression of MYH1 and MYH2 (which encode fast-twitch fiber types, ~87%). These expression levels were observed in LD muscle sampled three weeks before expected calving, with differences noted between high and low muscle depth groups. However, because bulk RNA-seq does not provide cell type resolution, these findings could not determine the precise proportions of myofiber subtypes or their functional implications.

In the study performed by Wang et al. [12], from which we sourced the scRNA-seq data as a reference for our study, the fractions of myofibers identified through scRNA-seq in the LD muscle were 57.21% for type I, 33.81% for type IIa, and 3.07% for type IIx. The higher proportions of type I myofiber in their study, compared to the proportion identified in this study is likely related to the age of the animals (relatively younger in Wang et al.’s [12] paper, ~4 months old). As demonstrated by Zhang et al. [15], the expression of type I in bovines reduces significantly between 3 to 36 months of age. In the bulk RNA-seq used in the deconvolution, the Holstein cows were at least 36 months of age. Also, Wang et al. [12] reported that the lower abundance of type IIx fibers was likely due to their larger size, as larger fragments were more prone to removal during sample processing.

### 4.5. Study Limitations and Technical Considerations

The discrepancies in muscle cell proportions may be partly attributed to methodological differences in the preparation of input samples, as scRNA-seq tends to exclude some myofibers due to their larger diameter, whereas snRNA-seq captures nuclei from all cell types, including those from larger myofibers, leading to a more comprehensive representation of muscle composition and potentially different estimated cell proportions. This underscores a key technical limitation of using scRNA-seq for analyzing myofibers. Large myofibers are frequently filtered out during the tissue dissociation and cell preparation steps required for scRNA-seq, which may compromise the results by underestimating certain fiber types, particularly the larger type IIx fibers. This technical bias could lead to skewed reference datasets that do not accurately represent the true cellular composition of muscle tissue. Future studies should consider employing snRNA-seq approaches, which can better capture nuclei from large myofibers and provide a more comprehensive representation of muscle fiber composition. Additionally, standardization of scRNA-seq preprocessing protocols (e.g., digestion steps) might help reduce technical biases during cell capture, thereby improving consistency across breeds and enabling more accurate comparisons of muscle composition.

The limited number of type IIx cells (10 cells) in the reference dataset represents a known limitation for deconvolution analysis, as rare cell types with insufficient reference cells often exhibit reduced signature matrix reliability and increased estimation variability across methods [42,43,44]. Recent methodological advances, such as hierarchical deconvolution [43], were specifically developed to address this challenge by employing hierarchical approaches to improve rare cell type estimation under limited reference conditions and should be explored in future studies. Another fundamental limitation of this study is the absence of ground truth single-cell RNA-seq data from Holstein cows to validate our deconvolution results. Without breed-matched reference data, we cannot definitively determine which deconvolution method or reference breed provides the most accurate estimates of myofiber proportions in Holstein muscle tissue. This limitation is critical for interpreting our findings and underscores the need for future studies to generate Holstein-specific single-cell reference data to establish definitive benchmarks for deconvolution accuracy assessment. Similarly, the age disparity between the reference animals (4-month-old calves in the Wang et al. [12] study) and the bulk RNA-seq samples (multiparous cows) is also a potential limitation of this study, as gene expression patterns can vary significantly between developmental stages. Another technical consideration that may have influenced our results was the use of log normalization for both input datasets. Since the reference data were in a log-normalized scale, we transformed the bulk RNA-seq data to the same scale to perform the deconvolution analysis. However, this transformation could introduce biases that impact the results. Specifically, log transformation compresses the range of high-expression values while amplifying lower expression values, which could affect the accuracy of the estimated cell proportions. Future studies should investigate the impact of different normalization strategies on deconvolution outcomes and develop standardized protocols for data preprocessing that minimize technical biases while preserving biological signals.

## 5. Implications for Future Research

The findings of this study extend beyond muscle fiber type analysis and have broader implications for the application of deconvolution methods in livestock genomics. The demonstration that breed-specific reference data significantly impact deconvolution outcomes suggests that similar effects may be observed in other tissues and cell types. Based on these findings, several recommendations can be made for future deconvolution analyses in livestock genomics. Researchers should carefully consider the genetic relationship between reference and target populations and, when possible, use reference datasets from the same breed or closely related breeds. When breed-matched reference datasets are not available, one option could be to validate results using multiple reference datasets from different breeds to assess the robustness of their findings. Additionally, reference and target datasets should be matched for developmental stage and physiological condition to minimize confounding effects, and multiple deconvolution methods should be employed to assess the consistency of results across different algorithmic approaches.

## 6. Conclusions

To the best of our knowledge, this is the first study to show that the cattle breed used in single-cell RNA-seq reference data significantly impacts deconvolution analyses for muscle fiber type estimation. In this context, CIBERSORTx and DeconvR-RLM seemed to demonstrate superior performance, providing biologically plausible estimates of muscle fiber composition (90–95% total myofiber content) with appropriate sensitivity to breed-specific variations. Notably, both methods also produced estimates that are consistent with known muscle fiber biology, further supporting their reliability and relevance for muscle deconvolution analyses. Given the complexity of breed-specific effects and current limitations in available reference datasets, we conclude that cell type proportion estimates should be determined using breed-specific references for accuracy in deconvolution approaches. Despite these promising findings, some limitations must be acknowledged. The reference dataset likely underrepresents large myofibers due to technical constraints of scRNA-seq, such as tissue dissociation bias. The small number of type IIx cells in the reference reduces estimation reliability for this fiber type. Moreover, the absence of breed- and age-matched single-cell data from Holstein cows, along with the use of log normalization, may have introduced biases in the estimated cell proportions. Future studies should prioritize the generation of single-cell RNA-seq data from the same populations used for bulk RNA-seq analysis to establish robust, population-specific reference datasets. Alternatively, developing comprehensive multiple-breed/inter-species reference datasets that capture the full spectrum of genetic diversity could support OneHealth initiatives by enabling cross-species comparative analyses and leveraging broader biological knowledge. Moreover, these advancements have important implications for government agencies and policymakers, as more precise muscle composition profiling can inform breeding programs, livestock management strategies, and sustainable agricultural policies aimed at improving animal health and productivity.

## Data Availability

The original contributions presented in this study are included in the article. Further inquiries can be directed to the corresponding author.
